# Supplementary material for: Kainate receptor channel opening and gating mechanism
Source: Nature. 2024 May 22;630(8017):762–8. doi: 10.1038/s41586-024-07475-0 (PMC11186766; doi:10.1038/s41586-024-07475-0)
Supplement: Supplementary file 1 — Sequence alignment of KAR subunits. Boundaries of ATD, LBD and TMD for GluK2 are depicted with differently coloured rectangles above the sequences. Glycosylation sites are indicated by red boxes and labelled. Glycosylation sites involved in ConA binding are labelled with red stars. [file 41586_2024_7475_MOESM1_ESM.pdf]

nature portfolio

https://doi.org/10.1038/s41586-024-07475-0

Supplementary information

Kainate receptor channel opening and gating mechanism

In the format provided by the authors and unedited

```
                                                                    N67   N73
                                        ATD
GluK2    (1) -MKIISPVLSNLVFSRSIKVLLCLLWIGYSQGTTHVLRFGGIFEYVE---SGPMGAEELAFRFAVNTINRNRTLLPNTTLTYDTQKINLYDSFEASKKAC
GluK1    (1) MERSTVLIQPGLWTRDTSWTLLYFLCYILPQTSPQVLRIGGIFETVE---NEPVNVEELAFKFAVTSINRNRTLMPNTTLTYDIQRINLFDSFEASRRAC
GluK3    (1) -MTAPWRRLRSLVWEYWAGFLVCAFWIPDSRGMPHVIRIGGIFEYADGPNAQVMNAEEHAFRFSANIINRNRTLLPNTTLTYDIQRIHFHDSFEATKKAC
GluK4    (1) ------------MPRVSAPLVLLPAWLLMVACSPHSLRIAAILDDPM----ECSRGERLSITLAKNRINRAPERLGKAKVEVDIFELLRDSEYETAETMC
GluK5    (1) ----------------MPAELLLLLIVAFANPSCQVLSSLRMAAILDD-QTVCGRGERLALALAREQINGIIEVPAKARVEVDIFELQRDSQYETTDTMC

                                        ATD
GluK2   (97) DQLSLGVAAIFGPSHS-SSANAVQSICNALGVPHIQTR-WKHQVSDNKDSFYVSLYPDFSSLSRAILDLVQFFKWKTVTVVYDDSTGLIRLQELIKAPSR
GluK1   (98) DQLALGVAALFGPSHS-SSVSAVQSICNALEVPHIQTR-WKHPSVDSRDLFYINLYPDYAAISRAVLDLVLYYNWKTVTVVYEDSTGLIRLQELIKAPSR
GluK3  (100) DQLALGVVAIFGPSQG-SCTNAVQSICNALEVPHIQLR-WKHHPLDNKDTFYVNLYPDYASLSHAILDLVQSLKWRSATVVYDDSTGLIRLQELIMAPSR
GluK4   (85) QILPKGVVAVLGPSSSPASSSIISNICGEKEVPHFKVAPEEFVRFQLQRFTTLNLHPSNTDISVAVAGILNFFNCTTACLICAKAECLLNLEKLLRQFLI
GluK5   (84) QILPKGVVSVLGPSSSPASASTVSHICGEKEIPHIKVGPEETPRLQYLRFASVSLYPSNEDVSLAVSRILKSFNYPSASLICAKAECLLRLEELVRGFLI

                                                                                     N275
                                        ATD
GluK2  (195) YNLRLKIRQLPADTKDAKPLLKEMKRGKEFHVIFDCSHEMAAGILKQALAMGMMTEYYHYIFTTLDLFALDVEPYRYSGVNMTGFRILNTENTQVSSIIE
GluK1  (196) YNIKIKIRQLPPANKDAKPLLKEMKKSKEFYVIFDCSHETAAEILKQILFMGMMTEYYHYFFTTLDLFALDLELYRYSGVNMTGFRKLNIDNPHVSSIIE
GluK3  (198) YNIRLKIRQLPIDSDDSRPLLKEMKRGREFRIIFDCSHTMAAQILKQAMAMGMMTEYYHFIFTTLDLYALDLEPYRYSGVNLTGFRILNVDNAHVSAIVE
GluK4  (185) SKDTLSVRMLD-DTRDPTPLLKEIRDDKTATIIIHANASMSHTILLKAAELGMVSAYYTYIFTNLEFSLQRMDSLVDDRVNILGFSIFNQSHAFFQEFSQ
GluK5  (184) SKETLSVRMLD-DSRDPTPLLKEIRDDKVSTIIIDANASISHLVLRKASELGMTSAFYKYILTTMDFPILHLDGIVEDSSNILGFSMFNTSHPFYPEFVR

                                                                                           N378
                                        ATD                                                 *
GluK2  (295) KWSMERLQAPPKPDSGLLDGFMTTDAALMYDAVHVVSVAVQQFP---QMTVSSLQCNRHKPWRFGTRFMSLIKEAHWEGLTGRITFNKTNGLRTDFDLDV
GluK1  (296) KWSMERLQAPPRPETGLLDGMMTTEAALMYDAVYMVAIASHRAS---QLTVSSLQCHRHKPCALGPRFMNLIKEARWDGLTGRITFNKTDGLRKDFDLDI
GluK3  (298) KWSMERLQAAPRAESGLLDGVMMTDAALLYDAVHIVSVCYQRAS---QMTVNSLQCHRHKPWRFGGRFMNFIKEAQWEGLTGRIVFNKTSGLRTDFDLDI
GluK4  (284) SLNQSWQENCDHVPFTG--PALS--SALLFDAVYAVVTAVQELNRSQEIGVKPLSCGSAQIWQHGTSLMNYLRMVELEGLTGHIEFN-SKGQRSNYALKI
GluK5  (283) SLNMSWRENCEASTYPG--PALS--AALMFDAVHVVVSAVRELNRSQEIGVKPLACTSANIWPHGTSLMNYLRMVEYDGLTGRVEFN-SKGQRTNYTLRI

                                            N412       N423   N430
                      ATD                                              LBD-S1
GluK2  (392) ISLKEEGLE---------------KIGTWDPASGLNMTESQKGKPANITDSLSNRSLIVTTILEEPYVLFKKSDKPLYGNDRFEGYCIDLLRELSTILGF
GluK1  (393) ISLKEEGTEKASGEVSKHLYKVWKKIGIWNSNSGLNMTDGNRDRSNNITDSLANRTLIVTTILEEPYVMYRKSDKPLYGNDRFEAYCLDLLKELSNILGF
GluK3  (395) ISLKEDGLE---------------KVGVWSPADGLNITEVAKGRGPNVTDSLTNRSLIVTTLLEEPFVMFRKSDRTLYGNDRFEGYCIDLLKELAHILGF
GluK4  (379) LQFTRNGFR---------------QIGQWHVAEGLSMD--SRLYASNISDSLFNTTLVVTTILENPYLMLKGNHQDMEGNDRYEGFCVDMLKELAEILRF
GluK5  (378) LEKSRQGHR---------------EIGVWYSNRTLAMN--ATTLDINLSQTLANKTLVVTTILENPYVMRRPNFQALSGNERFEGFCVDMLRELAELLRF

                                                                                  N546
                                     LBD-S1                                         *        TMD-M1
GluK2  (477) TYEIRLVEDGKYGAQDDVNGQWNGMVRELIDHKADLAVAPLAITYVREKVIDFSKPFMTLGISILYRKPNGTNPGVFSFLNPLSPDIWMYILLAYLGVSC
GluK1  (493) LYDVKLVPDGKYGAQND-KGEWNGMVKELIDHRADLAVAPLTITYVREKVIDFSKPFMTLGISILYRKPNGTNPGVFSFLNPLSPDIWMYVLLACLGVSC
GluK3  (480) SYEIRLVEDGKYGAQDD-KGQWNGMVKELIDHKADLAVAPLTITHVREKAIDFSKPFMTLGVSILYRKPNGTNPSVFSFLNPLSPDIWMYVLLAYLGVSC
GluK4  (462) NYKIRLVGDGVYGVPEA-NGTWTGMVGELIARKADLAVAGLTITAEREKVIDFSKPFMTLGISILYRVHMGRRPGYFSSLDPFSPGVWLFMLLAYLAVSC
GluK5  (461) RYRLRLVEDGLYGAPEP-NGSWTGMVGELINRKADLAVAAFTITAEREKVIDFSKPFMTLGISILYRVHMGRKPGYFSFLDPFSPAVWLFMLLAYLAVSC

        TMD-M1                          TMD-M2                    TMD-M3
GluK2  (577) VLFVIARFSPYEWYNPHPCN-PDSDVVENNFTLLNSFWFGVGALMQQGSELMPKALSTRIVGGIWWFFTLIIISSYTANLAAFLTVERMESPIDSADDLA
GluK1  (592) VLFVIARFTPYEWYNPHPCN-PDSDVVENNFTLLNSFWFGVGALMQQGSELMPKALSTRIVGGIWWFFTLIIISSYTANLAAFLTVERMESPIDSADDLA
GluK3  (579) VLFVIARFSPYEWYDAHPCN-PGSEVVENNFTLLNSFWFGMGSLMQQGSELMPKALSTRIIGGIWWFFTLIIISSYTANLAAFLTVERMESPIDSADDLA
GluK4  (561) VLFLVARLTPYEWYSPHPCAQGRCNLLVNQYSLGNSLWFPVGGFMQQGSTIAPRALSTRCVSGVWWAFTLIIISSYTANLAAFLTVQRMEVPIESVDDLA
GluK5  (560) VLFLAARLSPYEWYNPHPCLRARPHILENQYTLGNSLWFPVGGFMQQGSEIMPRALSTRCVSGVWWAFTLIIISSYTANLAAFLTVQRMEVPVESADDLA

                                                                                   N751
                                        LBD-S2                                       *
GluK2  (676) KQTKIEYGAVEDGATMTFFKKSKISTYDKMWAFMSSRRQSVLVKSNEEGIQRVLTSDYAFLMESTTIEFVTQRNCNLTQIGGLIDSKGYGVGTPMGSPYR
GluK1  (691) KQTKIEYGAVRDGSTMTFFKKSKISTYEKMWAFMSSRQQSALVKNSDEGIQRVLTTDYALLMESTSIEYVTQRNCNLTQIGGLIDSKGYGVGTPIGSPYR
GluK3  (678) KQTKIEYGAVKDGATMTFFKKSKISTFEKMWAFMSSK-PSALVKNNEEGIQRTLTADYALLMESTTIEYITQRNCNLTQIGGLIDSKGYGIGTPMGSPYR
GluK4  (661) DQTAIEYGTIHGGSSMTFFQNSRYQTYQRMWNYMYSKQPSVFVKSTEEGIARVLNSNYAFLLESTMNEYYRQRNCNLTQIGGLLDTKGYGIGMPVGSVFR
GluK5  (660) DQTNIEYGTIHAGSTMTFFQNSRYQTYQRMWNYMQSKQPSVFVKSTEEGIARVLNSRYAFLLESTMNEYHRRLNCNLTQIGGLLDTKGYGIGMPLGSPFR

              LBD-S2                          TMD-M4
GluK2  (776) DKITIAILQLQEEGKLHMMKEKWWRGNGCPEEESKEASALGVQNIGGIFIVLAAGLVLSVFVAVGEFLYKSKKNAQLEKRSFCS----------------
GluK1  (791) DKITIAILQLQEEGKLHMMKEKWWRGNGCPEEDSKEASALGVENIGGIFIVLAAGLVLSVFVAIGEFLYKSRKNNDVEQKGKSSRLRFYFRNKVRFHGSK
GluK3  (777) DKITIAILQLQEEDKLHIMKEKWWRGSGCPEEENKEASALGIQKIGGIFIVLAAGLVLSVLVAVGEFIYKLRKTAEREQRSFCS----------------
GluK4  (761) DEFDLAILQLQENNRLEILKRKWWEGGKCPKEEDHRAKGLGMENIGGIFVVLICGLIVAIFMAMLEFLWTLRHS-EASEVSVCQ----------------
GluK5  (760) DEITLAILQLQENNRLEILKRKWWEGGRCPKEEDHRAKGLGMENIGGIFVVLICGLIIAVFVAVMEFIWSTRRSAESEEVSVCQ----------------
```

Supplementary Figure 1. Sequence alignment of KAR subunits. Boundaries of ATD, LBD and TMD for GluK2 are depicted by different color rectangles above the sequences. Glycosylation sites are indicated by red boxes and labeled. Glycosylation sites involved in ConA binding are also labeled by red stars.
